# Arginine Thiazolidine Carboxylate Stimulates Insulin Secretion through Production of Ca^2+^-Mobilizing Second Messengers NAADP and cADPR in Pancreatic Islets

**DOI:** 10.1371/journal.pone.0134962

**Published:** 2015-08-06

**Authors:** Dae-Ryoung Park, Asif Iqbal Shawl, Tae-Geun Ha, Kwang-Hyun Park, Seon-Young Kim, Uh-Hyun Kim

**Affiliations:** 1 Department of Biochemistry, Chonbuk National University Medical School, Jeonju, Korea; 2 National Creative Research Laboratory for Ca^2+^ Signaling Network, Chonbuk National University, Jeonju, Korea; 3 Institute of Cardiovascular Research, Chonbuk National University, Jeonju, Korea; College of Tropical Agriculture and Human Resources, University of Hawaii, UNITED STATES

## Abstract

Oxothiazolidine carboxylic acid is a prodrug of cysteine that acts as an anti-diabetic agent via insulin secretion and the formation of the Ca^2+^-mobilizing second messenger, cyclic ADP-ribose (cADPR). Here we show that a hybrid compound, arginine thiazolidine carboxylate (ATC), increases cytoplasmic Ca^2+^ in pancreatic β-cells, and that the ATC-induced Ca^2+^ signals result from the sequential formation of two Ca^2+^-mobilizing second messengers: nicotinic acid adenine dinucleotide phosphate (NAADP) and cADPR. Our data demonstrate that ATC has potent insulin-releasing properties, due to the additive action of its two components; thiazolidine carboxylate (TC) and _L_-arginine. TC increases glutathione (GSH) levels, resulting in cAMP production, followed by a cascade pathway of NAADP/nitric oxide (NO)/cGMP/cADPR synthesis. _L_-arginine serves as the substrate for NO synthase (NOS), which results in cADPR synthesis via cGMP formation. Neuronal NOS is specifically activated in pancreatic β-cells upon ATC treatment. These results suggest that ATC is an ideal candidate as an anti-diabetic, capable of modulating the physiological Ca^2+^ signalling pathway to stimulate insulin secretion.

## Introduction

Thiazolidines are a class of heterocyclic organic compounds that has a 5-member saturated ring with a thioether group and an amine group in the 1 and 3 positions, respectively. It is a sulfur analog of oxazolidine [1]. We have previously demonstrated that a thiazolidine analog, oxothiazolidine carboxylic acid (OTC), enhances insulin secretion in pancreatic islets [2]. The mechanism by which OTC enhances insulin secretion was explained by its ability to increase intracellular glutathione levels as a prodrug of cysteine, a rate-limiting precursor in glutathione synthesis. An increase in intracellular reducing power was able to induce CD38 dimerization and internalization, which resulted in the production of cyclic ADP-ribose (cADPR), a Ca^2+^-mobilizing messenger [2].

_L_-Arginine potentiates glucose-induced insulin release [3], and arginine-derived nitric oxide (NO) has been suggested as a mediator in insulin secretion stimulated by arginine. It was shown that the potentiation of glucose-induced insulin release by arginine is a Ca^2+^-dependent mechanism, which results from membrane depolarization and the rise of cytoplasmic Ca^2+^ in β-cells [4]. NO was first discovered as a mediator in vascular smooth muscle relaxation, where it induces a decrease in intracellular free calcium [Ca^2+^]_i_ [5], but accumulating evidence demonstrated that treatment with NO or NO donors elicits a rise in [Ca^2+^]_i_ in a variety of cells, including pancreatic β-cells [6–8]. Furthermore, the regulation of insulin release by arginine has been reported to be deficient in patients with type 2 diabetes mellitus [9]. These findings indicate that arginine plays a beneficial role in glucose homeostasis by targeting β-cells.

ADP-ribosyl cyclases (ARCs) catalyze the synthesis and hydrolysis of two Ca^2+^-mobilizing second messengers: cADPR and nicotinic acid adenine dinucleotide phosphate (NAADP) [10–11]. ARC was first purified and cloned from the ovotestis of *Aplysia californica* [12,13]. Later, CD38, a T cell surface antigen, was found to be an ARC homolog [14], and it has been known to be a mammalian prototype of ARCs [15].

Since the first demonstration of the role of cADPR in pancreatic islets [16], its role in insulin secretion was confirmed by the demonstration of CD38-induced intracellular cADPR production [17]. Mounting evidence showed that NAADP also played a key role in the endocrine pancreas [18–20]. Our previous study demonstrated that glucagon-like peptide-1 (GLP-1)-mediated Ca^2+^ signals in insulin secretion from pancreatic β-cells is a cooperative process between the actions of cADPR and NAADP [21]. The initial phase of the GLP-1-activated Ca^2+^ signals is due to NAADP-mediated Ca^2+^ mobilization from acidic stores, while the second, maintained phase is attributable to the cADPR-mediated Ca^2+^ signal mediated through the endoplasmic reticulum. In support of these data, β-cells isolated from CD38^-/-^ mice showed reduced production of NAADP and cADPR after activation by GLP-1, indicating a partial dependence on CD38.

In the present study, we synthesized arginine thiazolidine carboxylate (ATC), a hybrid compound derived from a prodrug of cysteine, and examined its effects on insulin secretion and Ca^2+^ signaling in pancreatic islets. We found that the hybrid molecule displayed higher potency in inducing insulin secretion as well as NAADP and cADPR production when compared to its analogs or precursor components.

## Materials and Methods

### Reagents

Dulbecco’s Modified Eagle’s medium (DMEM) containing low glucose, and antibiotics were from GIBCO (Grand Island, NY, USA). (*R*p)-8-pCPT-cGMP-S was purchased from Calbiochem (Darmstadt, Germany), All other reagents were obtained from Sigma (Sigma Aldrich, St Louis, USA). Recombinant nicotinic acid mononucleotide adenylyltransferase (NMN-AT) was a gift from Dr. S. W. Suh [22].

### Synthesis of arginine thiazolidine carboxyiate

Thiazolidine-2-carboxylic acid or thiazolidine-4-carboxylic acid (0.94 mmol) was dissolved in methanol (25 mL) under stirring. The solution was stirred at room temperature for 10 min. During stirring _L_-arginine hydrochoride (0.94 mmol) in distilled water (5 mL) was added dropwise into flask. The reaction mixture was stirred at 50 °C until obtain clear solution. Methanol was removed by rotary evaporator. The obtained solid was dried under vacuum. The solid was suspended in acetone (100 mL) and stirred vigorously. The solid was filtered and dried under vacuum, to give arginine thiazolidine-2-carboxylate or arginine thizaolidine-4-carboxyliate as a white solid. ^1^H- and ^13^C-NMR spectra were recorded on Jeol 400 MHz spectrometer. Chemical shifts are shown in δ values (ppm). Arginine thizaolidine-2-carboxylate; Yield: 78.1%; ^1^H NMR (400 MHz, D_2_O) δ 5.03 (s, 1H), 3.67–3.61 (m, 2H), 3.56–3.50 (m, 1H), 3.13–3.08 (m, 4H), 1.79–1.72 (m, 2H), 1.63–1.44 (m, 2H); ^13^C NMR (100 MHz, D_2_O) δ 175.2, 172.7, 157.7, 63.4, 55.3, 50.4, 41.6, 30.6, 28.5, 24.9; ESI MS: m/z 308.1 (M + H)^+^. Arginine thizaolidine-4-carboxylate; Yield: 67.9%; ^1^H NMR (400 MHz, D_2_O) δ 4.22 (d, *J* = 9.76, 1H), 3.86 (d, *J* = 9.76, 1H), 3.62 (t, *J* = 5.84, 1H), 3.50 (t, *J* = 7.8, 1H), 3.16–3.09 (m, 3H), 2.69–2.64 (m, 1H), 1.79–1.74 (m, 2H), 1.63–1.54 (m, 2H); ^13^C NMR (100 MHz, D_2_O) δ 178.7, 175.5, 157.6, 68.2, 55.2, 53.4, 41.4, 37.2, 28.5, 24.7; ESI MS: m/z 308.1 (M + H)^+^.

### Animals

Mice with genetic background ICR were inbred in the Animal Facility of Chonbuk National University Medical School. CD38 knockout mice (*Cd38* ^-/-^; B6.129P2-Cd38^tm/Lud^) were purchased from Jackson Laboratory (Bar Harbor, ME). Male C57BL/KsJ-db/db mice (7 weeks old) were purchased from Clea Japan (Tokyo, Japan). All animal-based investigations were designed and performed in accordance with the Guide for the Care and Use of Laboratory Animals published by the National Institutes of Health (NIH Pub. No. 85–23, revised 1996) [23]. The entire project was reviewed and approved by the Institutional Animal Care and Use Committee of the Chonbuk National University Medical School (CBU 2013–0009).

### Preparation of islets

Pancreatic islets were isolated from ICR background mice, weighing 25–30 g, using a collagenase method, except Krebs-Ringer bicarbonate buffer (KRB buffer in mmol/L: 2 CaCl_2_, 2.8 glucose, 145 NaCl, 1.19 KCl, 2.54 MgCl_2_, 1.19 KH_2_PO_4,_ 5 NaHCO_3_, 20 HEPES, pH 7.3), was used instead of Krebs-Ringer buffer. Briefly, sacrifice animal by cervical dislocation. Make a V-incision starting at the genital area of the mouse. Pancreas was distended by infusion of KRB buffer containing 0.15 mg/ml type V collagenase through the bile duct. Incubate for 20–25 minutes at 37°C in water-bath. Islets were isolated, washed with KRB buffer, resuspend the pellet in ficoll and centrifuge, islets were handpicked to eliminate any remaining exocrine tissue and stabilized by culturing overnight at 37°C in a humidified incubator (95% air, 5% CO_2_) in low glucose (5 mmol/L) DMEM supplemented with 10% (v/v) fetal bovine serum (FBS), 100 units/ml penicillin G, and 100 μg/mL streptomycin (culture media).

### Measurement of [Ca^2+^]_i_

Dispersed β-cells obtained by trituration of islets with a pipette were seeded on confocal dishes and cultured in culture media. β-cells were identified on the basis of their large diameter and granular appearance. Adhered cells were washed with KRB buffer containing 0.1% BSA and loaded with 1 μM Fluo-3 AM (Molecular Probe, Eugene, OR) at 37°C for 30 min. After washing with KRB buffer containing 0.1% BSA, an appropriate amount of glucose was supplemented. Changes in [Ca^2+^]_i_ were determined at 488/530 nm excitation/emission by air-cooled argon laser system [24]. The emitted fluorescence at 530 nm was collected using a photomultiplier. One image every 3 sec was scanned using confocal microscope (Nikon, Japan). [Ca^2+^]_i_ calculation was performed by suing an equation given by Tsein *et al* [25], i.e. [Ca^2+^]_i_ = K_d_(F-F_min_)/(F_max_-F), where K_d_ is 450 nM for Fluo-3 and F is the observed fluorescence levels. Each tracing was calibrated for the maximal intensity (F_max_) by addition of 8 μmol/L ionomycin and for the minimal intensity (F_min_) by addition of 50 mmol/L EGTA at the end of each measurement. Fluorescence in β-cells was determined as described previously [21].

### Measurement of cAMP production

Levels of cAMP were determined in whole islets as described previously [26]. Batches of 40 islets were incubated for 15 min at 37°C in KR buffer supplemented with 0.1% BSA, 400 μmol/L ATC, 12 mmol/L glucose. cAMP content of acetylated samples was measured using a commercially available cAMP ELISA assay kit (Enzo Life Sciences, Farmingdale, NY, USA).

### Measurement of intracellular cADPR concentration

Cyclic enzymatic assay was performed to measure cADPR levels as described previously [27]. Briefly 40–50 islets were treated with 0.6 mol/L perchloric acid (PCA) under sonication. Precipitates were removed by centrifugation at 20,000 x *g* for 10 min at 4°C. To remove PCA, aqueous sample was mixed with a solution containing 1,1,2-trichlorotrifluoroethane and tri-*n*-octylamine in the ratio of 3:1. After centrifugation for 10 min at 1500 x *g*, the aqueous layer was collected and neutralized with 20 mmol/L sodium phosphate buffer (pH 8.0). To remove contaminating nucleotides, the samples were incubated overnight with 2.5 mmol/L MgCl_2_ and following enzymes in 20 mmol/L sodium phosphate buffer (pH 8.0) at 37°C: 0.44unit/mL nucleotide pyrophosphatase, 12.5 unit/ml alkaline phosphatase and 0.625 unit/ml NADase. Enzymes were removed by filtration using Centricon-3 filters. To convert cADPR to NAD^+^, the samples (0.1 mL/tube) were incubated with 50 μl of a cycling reagent containing 0.3 μg/ml *Aplysia* ADPR cyclase, 30 mmol/L nicotinamide, and 100 mmol/L sodium phosphate (pH 8.0), at room temperature for 15 min. The samples were further incubated with the cycling reagent (0.1 mL) containing 2% ethanol, 100 μg/mL alcohol dehydrogenase, 20 μmol/L resazurin, 10 μg/ml diaphorase, 10 μmol/L riboflavin 5-phosphate, 10 mmol/L nicotinamide, 0.1 mg/mL BSA and 100 mmol/L sodium phosphate (pH 8.0) for 2–4 hours at room temperature. An increase in resorufin fluorescence was measured at 544 nm excitation and 590 nm emission using a fluorescence microplate reader (Molecular Devices Corp., Spectra-Max GEMINI). Level of cADPR was measured using a cyclic enzymatic assay as described previously. For the experiment, *Neurospora crassa* NADase and *Aplysia* ARC were purified according to the methods described by Cho *et al* [28] and Lee *et al* [29], respectively.

### Measurement of intracellular NAADP concentration

Cyclic enzymatic assay was performed to measure NAADP levels as described previously [27]. Briefly 40–50 islets were treated with 0.6 mol/L PCA under sonication. Precipitates were removed by centrifugation at 20,000 x *g* for 10 min at 4°C. To remove PCA, aqueous sample was mixed with a solution containing 1,1,2-trichlorotrifluoroethane and tri-*n*-octylamine in the ratio of 3:1. After centrifugation for 10 min at 1500 x *g*, the aqueous layer was collected and neutralized with 20 mmol/L sodium phosphate buffer (pH 8.0). To remove contaminating nucleotides, the samples were incubated overnight with 2 mmol/L MgCl_2_, 1 mmol/L sodium fluoride and 0.1 mmol/L inorganic pyrophosphate and following enzymes in 20 mmol/L sodium phosphate buffer (pH 8.0) at 37°C: 2.5 unit/mL Apyrase, 0.16 mg/mL NMN-AT and 0.125 unit/mL NADase. Enzymes were removed by filtration using Centricon-3 filters. Unlike conversion of cADPR to NAD^+^, NAADP is first converted to nicotinic acid adenine dinucleotide (NAAD) by addition of 10 unit/ml of alkaline phosphatase overnight at 37°C. Enzymes were removed by filtration using Centricon-3 filters. NAAD is further converted to NAD by the 0.2 mg/mL NMN-AT. NMN-AT is a reversible enzyme and can use NAAD as well as NAD as substrate. NMN-AT converts NAAD to nicotinic acid mononucleotide (NAMN) and ATP in the presence of 0.5 mmol/L inorganic pyrophosphate (PPi). 0.2 mmol/L NMN is included which combines with ATP to form NAD in presence of 2 mmol/L MgCl_2_, 10 mmol/L nicotinamide, 2 mmol/L sodium fluoride and 100 mmol/L Tris-HCl (pH-8.0) at room temperature for 30 min. The samples were further incubated with the cycling reagent containing 2% ethanol, 100 μg/ml alcohol dehydrogenase, 20 μmol/L resazurin, 10 μg/mL diaphorase, 10 μmol/L riboflavin 5-phosphate, 10 mmol/L nicotinamide, 0.1 mg/mL BSA and 100 mmol/L sodium phosphate (pH 8.0) for 2–4 h at room temperature. An increase in resorufin fluorescence was measured at 544 nm excitation and 590 nm emission using a fluorescence microplate reader (Molecular Devices Corp., Spectra-Max GEMINI). Level of cADPR was measured using a cyclic enzymatic assay as described previously. For the experiment, *Neurospora crassa* NADase and *Aplysia* ARC were purified according to the methods described by Cho *et al* [28] and Lee *et al* [29], respectively.

### cGMP enzyme-linked immunoassay

Cultured isolated islets (25 islets/sample) were washed twice with fresh medium. Ice-cold HCl (final concentration 0.1 M) was added to stop the reaction. Samples were frozen and thawed three times, sonicated, and centrifuged at 500 rpm for 10 min. Supernatants were assayed for cGMP by enzyme-linked immunoassay kit (Cell Signaling Technology, Inc., Massachusetts, USA) [30].

### Nitrite measurement

Biologically produced NO is rapidly oxidized to nitrite and nitrate in aqueous solutions [31,32]. Nitrite concentrations in the cell-free culture supernatants, therefore, served as a reflection of NO production and were measured using a previously described colorimetric assay [33]. Following overnight incubation at 37°C in a humidified 95% air/5% CO_2_ atmosphere, nitrite concentration was measured from 30 islets in KR buffer with/without ATC. Briefly, 50-μl aliquots of supernatants dispensed into 96-well microtiter plates (flat bottom) (SPL, Life Sciences, Pocheon, Republic of Korea) were incubated with 100 ml of a 1:1 mixture of 1% sulfanilamide (Sigma Aldrich, St Louis, USA) in 30% acetic acid and 0.1% *N*-(1-naphthyl)ethylene-diamine dihydrochloride (Sigma Aldrich, St Louis, USA) in 60% acetic acid at room temperature. After 5 min, absorbance was measured at 570 nm using a micro plate reader (BioRad XMark, Microplate Spectrophotometer, Japan). Concentrations were determined from a linear standard curve obtained from serial concentrations (6.25–200 mM) of sodium nitrite (Sigma Aldrich, St Louis, USA) in working medium. Results of triplicate measurements were presented as means ± SD.

### Glucose Tolerance Test

Male C57BL/KsJ-db/db mice (7 weeks old) were purchased from Clea Japan (Tokyo, Japan). Mice were maintained in an environmentally controlled room with a 12-h light-dark cycle and were allowed free access to diet and a specific pathogen-free water. For glucose tolerance test, mice were fasted overnight and received single oral administration of various concentrations of ATC (0–40 mg/kg). Five h later, mice were injected with glucose (0.5 g/kg, intraperitoneally), and blood samples were taken at various time points (0–120 min). Blood glucose levels were measured by the glucose oxidase method using a glucose analyzer (Lifescan, Inc., Milpitas, CA), and serum insulin levels were determined using a radioimmunoassay kit (Amersham Biosciences, Inc.).

### Statistical analysis

Data represent means ± standard error of the mean (SEM) of at least three separate experiments. Statistical analysis was performed using Student's *t*-test or Repeated measures AVOVA. A value of *p* < 0.05 was considered significant.

## Results

### Design and synthesis of an anti-diabetic prodrug

The effects of arginine and thiazolidine carboxylic acid (TC) in promoting insulin secretion in pancreatic islets prompted us to develop an anti-diabetic hybrid prodrug (See the chemical structures; S1 Fig). L-arginine, a substrate for nitric oxide (NO) synthase, has been known to increase insulin secretion [3], and an analog of TC, oxothiazolidine-4-carboxylic acid (OTC), has also been known to enhance insulin secretion [2]. Before synthesizing a hybrid compound from the two precursor molecules, we compared the potency of two different TCs, thiazolidine-2-carboxylic acid (T2C) [34] and thiazolidine-4-carboxylic acid (T4C) [35], in their ability to generate intracellular glutathione (GSH) in islets, since intracellular GSH generation by TCs enhances insulin secretion [2]. GSH generation induced by T2C was higher than that by T4C (S2 Fig). Thus, we chose T2C for the synthesis of an arginine salt of thiazolidine-2-carboxylic acid (ATC), and used this ATC as a hybrid prodrug throughout the study.

### ATC displayed greater potency in inducing Ca^2+^ signaling and insulin secretion when compared to precursor and analog molecules

**Fig 1:**
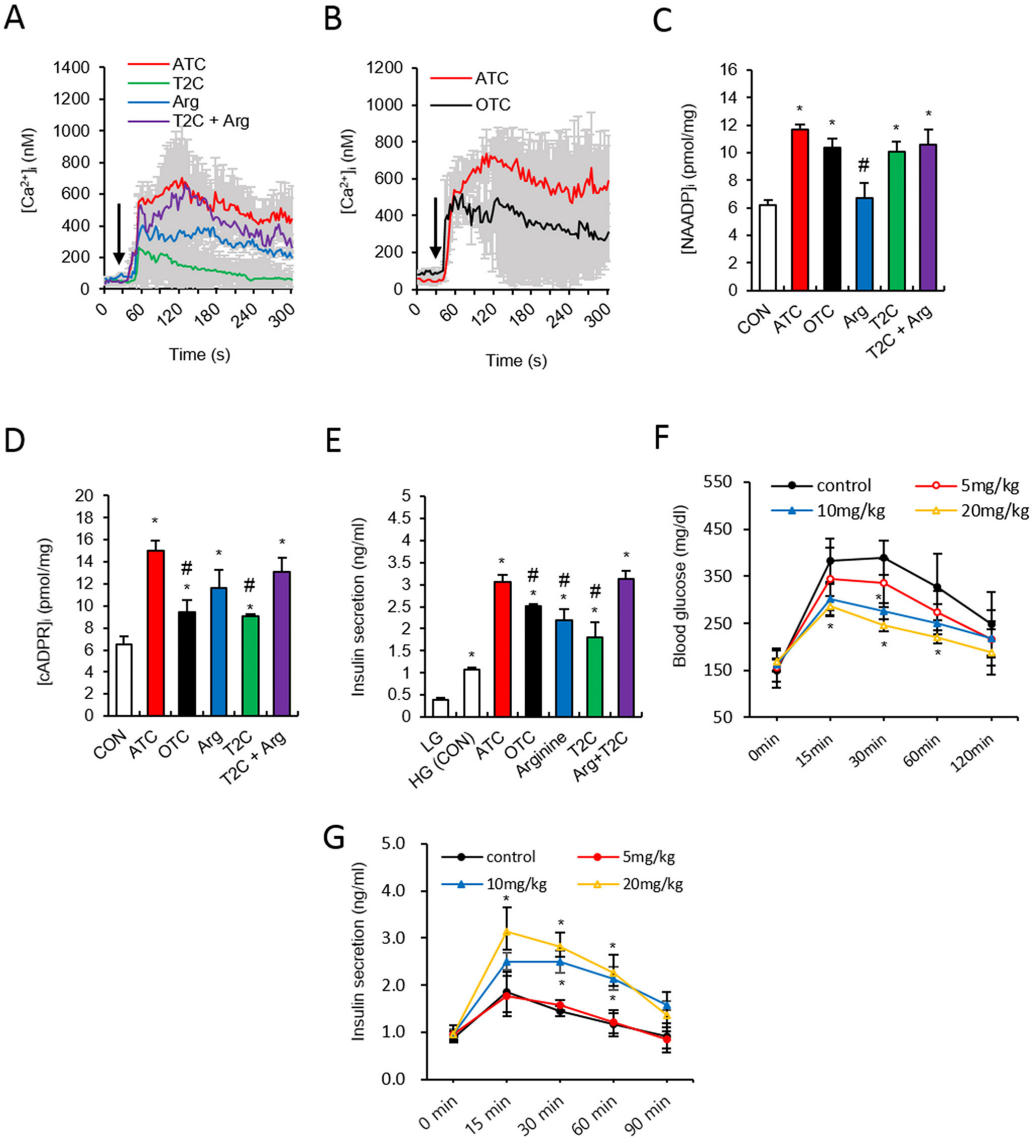
ATC induces insulin secretion via cADPR/NAADP-dependent Ca^2+^ signals in pancreatic β cell. **(A**) Representative tracing of the Ca^2+^ response to ATC (400 μM), Arginine (400 μM) (AG), Thiazolidine-2-carboxylic acid (400 μM) (T2C) and T2C + Arg treatments. **(B)** Representative tracing of the Ca^2+^ response to ATC (400 μM) and OTC (1 mM) treatments. **(C)** Comparisons of NAADP formation among ATC, OTC, Arg, T2C and T2C + AG treatment. **(D)** Comparisons of cADPR formation among ATC, OTC, Arg, T2C and T2C + Arg treatment. **(E)** Comparisons of insulin secretion among ATC, OTC, Arg, T2C and T2C + AG treatment. **(F)** Blood glucose levels in vehicle (*closed circle*, *n* = 5)- and ATC (5 mg/kg; *open circle*, *n* = 5, 10 mg/kg; *closed triangle*, *n* = 5, 20 mg/kg; *open triangle*, *n* = 5)-treated *db*/*db* mice following intraperitoneal injection of glucose after overnight fasting. **(G)** Plasma insulin levels during intraperitoneal glucose tolerance testing in vehicle (*closed circle*, *n* = 5)- and ATC (5 mg/kg; *open circle*, *n* = 5, 10 mg/kg; *closed triangle*, *n* = 5, 20 mg/kg; *open triangle*, *n* = 5)-treated *db*/*db* mice. *, P<0.05 versus CON level. #, P<0.05 versus ATC treated level. All data are expressed as the Mean ± SEM.

To obtain a dose-response curve of ATC, we performed experiments of cADPR and NAADP production with various dose of ATC (100, 400, 700 μM and 1 mM). Since our data showed that 400 μM ATC resulted in a plateau of cADPR and NAADP production (S3 Fig), we used 400 μM ATC in all experiments through the present work. In order to compare the potency of precursors and hybrid compounds, [Ca^2+^]_i_ was measured in islets following treatment with the following agents: ATC (400 μM), OTC (1 mM), T2C (400 μM), arginine (400 μM), and arginine plus T2C. T2C and arginine induced a Ca^2+^ rise with less potency when compared to ATC. (Fig 1A). Treatment of pancreatic β-cells with 400 μM ATC induced a long lasting Ca^2+^ increase, which was also higher in amplitude, even when compared to higher concentration of OTC (1 mM) (Fig 1B).

Based on our previous findings that OTC induces CD38-mediated Ca^2+^ signalling, resulting in insulin secretion [2], we examined whether the agents induced NAADP and cADPR production. ATC strongly induced the production of both NAADP and cADPR, compared to OTC, T2C, arginine, or arginine plus T2C (Fig 1C and 1D), indicating that ATC is a potent inducer of NAADP and cADPR formation, resulting in a rise in Ca^2+^. Arginine produced only cADPR and no NAADP, while other agents induced the production of both messengers to degrees comparable to their Ca^2+^ signalling potency. ATC-induced insulin secretion was significantly higher than those induced by OTC, T2C, or arginine, and it was comparable to that of arginine plus T2C (Fig 1E). Finally, to examine the effects of ATC on glucose levels *in vivo*, we treated mice with 5, 10, and 20 mg/kg ATC. Plasma glucose levels in mice subjected to a glucose challenge after an overnight fast were significantly ameliorated by treatment with 10 mg/kg ATC or above, and plasma insulin levels were significantly increased (Fig 1F and 1G).

### ATC induces Ca^2+^ rise via NAADP and cADPR formation

**Fig 2:**
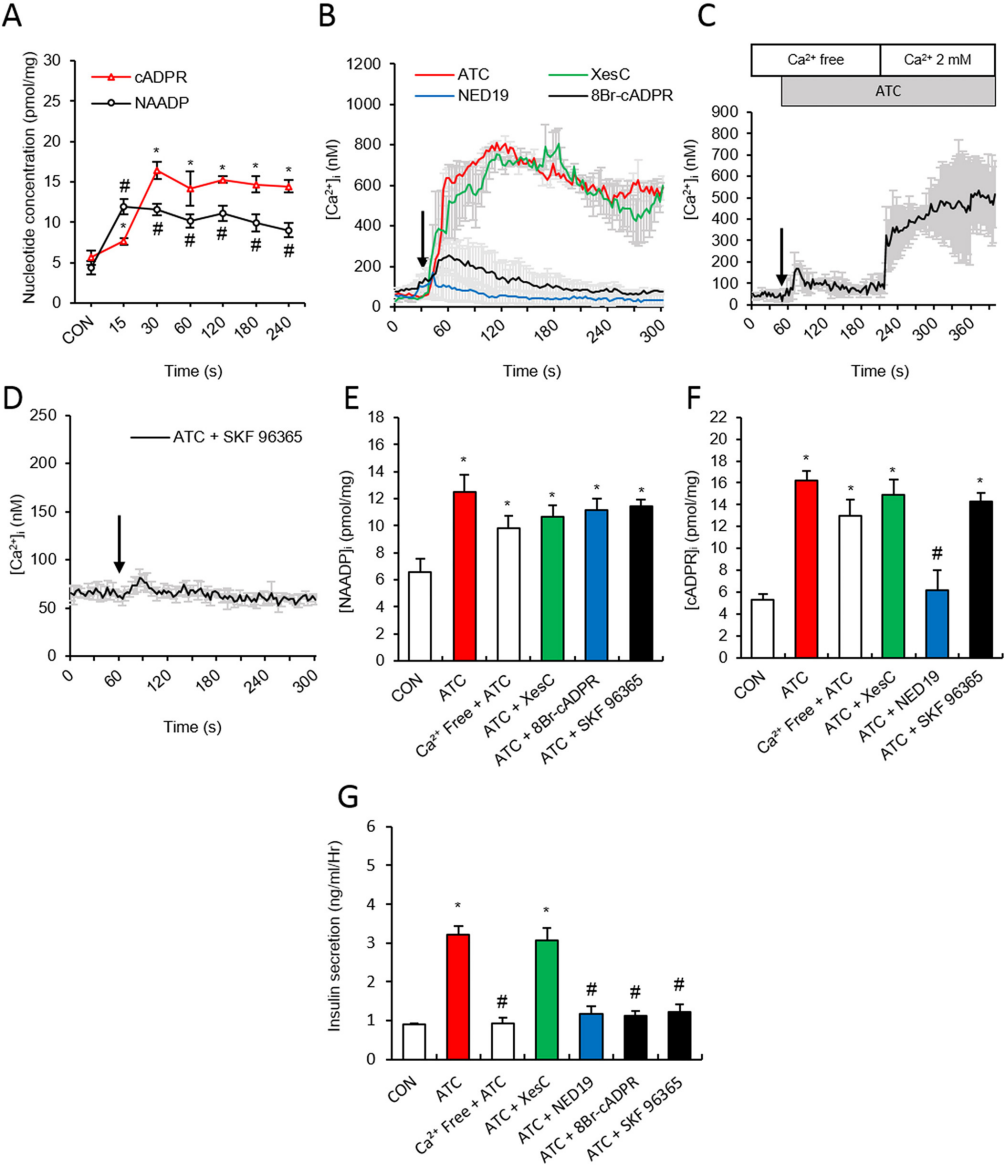
ATC-induced NAADP and cADPR formation and involvement of SOCE in ATC-induced Ca^2+^ signaling in pancreatic β cell. **(A)** Time course of NAADP and cADPR production following ATC treatment **(B)** Effect of Ca^2+^ second messenger inhibitors on ATC-induced Ca^2+^ signals. XesC (2 μM), Ned19 (100 μM) and 8-Br-cADPR (100 μM) were used. **(C)** Representative tracings of the Ca^2+^ response to ATC in the absence and presence of extracellular Ca^2+^. **(D)** Representative tracings of the Ca^2+^ response to ATC in the presence of SKF 96365 (10 μM) **(E and F)** Effect of Ca^2+^ second messenger inhibitors on ATC-induced cADPR and NAADP formation. **(G)** Effect of Ca^2+^ second messenger inhibitors on ATC-induced insulin secretion. *, P<0.05 versus CON level. #, P<0.05 versus ATC treated level. All data are expressed as the Mean ± SEM.

Because ATC was found to induce both NAADP and cADPR production, we examined the time course for the production of both Ca^2+^ second messengers. NAADP formation, reaching its plateau at 15 sec, preceded cADPR formation, with its peak at 30 sec (Fig 2A). The ATC-mediated signalling pathway was examined by determining changes in ATC-induced [Ca^2+^]_i,_ and NAADP and cADPR levels under various conditions. The ATC-induced Ca^2+^ rise was abolished by pretreatment of cells with Ned19 (100 μM), a NAADP receptor antagonist, but not with XeC (2 μM), an IP_3_ receptor blocker. Pretreatment of cells with a cADPR antagonist, 8-Br-cADPR, only affected the sustained Ca^2+^ rise, while the initial rise remained intact (Fig 2B). Similarly, pretreatment with high concentration of ryanodine (20 μM) as a ryanodine receptor blocker resulted in an inhibition of the later phase of ATC-induced Ca^2+^ signals (S4 Fig). We supposed the existence of a store-operated Ca^2+^ entry (SOCE) mechanism in ATC-induced sustained Ca^2+^ signaling. To test this notion, we treated pancreatic islets with ATC in Ca^2+^ free condition and found only a Ca^2+^ transient, presumably due to Ca^2+^ mobilization via the actions of NAADP and cADPR. The addition of extracellular Ca^2+^ resulted in sustained Ca^2+^ signals, indicating that the depletion of Ca^2+^ stores induced Ca^2+^ entry (Fig 2C). SOCE involvement in ATC-induced sustained Ca^2+^ signalling was confirmed by evidence that the ATC-mediated sustained Ca^2+^ rise was blocked by pretreatment of cells with SK96365 (100 μM), a SOCE blocker (Fig 2D). NAADP formation was not affected by preincubation with 8-Br cADPR, an antagonistic analogue of cADPR, confirming that cADPR follows NAADP formation. Ca^2+^ free conditions, XeC, IP_3_ receptor antagonists, and SKF96365 did not block ATC-induced NAADP formation (Fig 2E). On the other hand, ATC-induced cADPR formation was blocked by the NAADP receptor antagonist, Ned19. However, XeC, IP_3_ receptor antagonists, SKF96365, SOCE blockers, and Ca^2+^ free conditions did not affect cADPR formation (Fig 2F). ATC-induced insulin secretion was blocked by Ned19, 8-Br-cADPR, extracellular Ca^2+^ free condition, and SKF96365, though XeC did not affect ATC-induced insulin release (Fig 2G). Together, these results suggest that ATC induces the sequential production of NAADP and cADPR, followed by SOCE for sustained Ca^2+^ signalling, ultimately resulting in insulin secretion.

### ATC-induced NAADP and cADPR formation is dependent on cAMP

**Fig 3:**
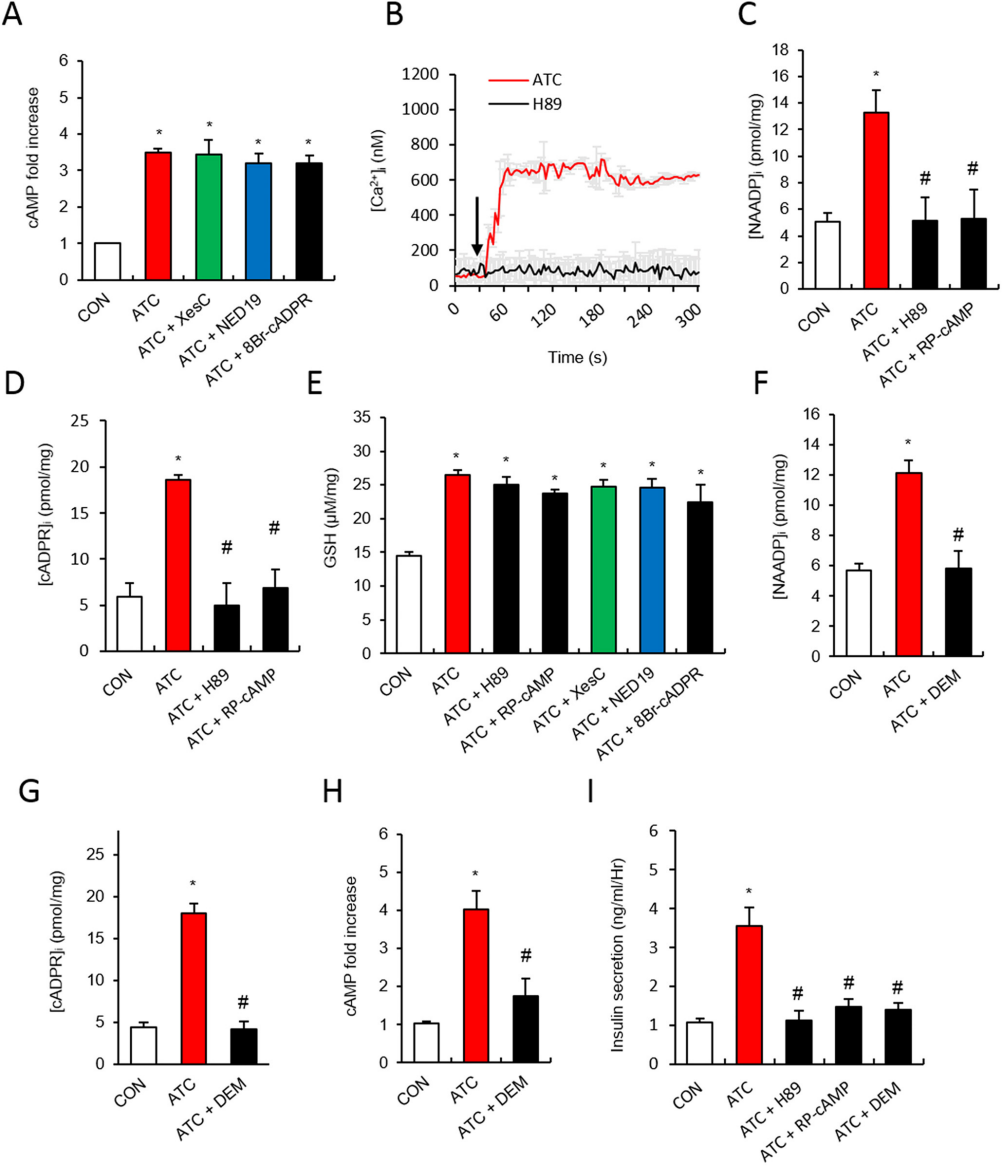
ATC-induced NAADP and cADPR formation in a cAMP-dependent manner in pancreatic β cell. **(A)** Effect of H89 (10 μM) on ATC-induced Ca^2+^ signals. **(B)** Effect of Ca^2+^ second messenger inhibitors on ATC-induced cAMP formation. **(C and D)** Effect of H89 and Rp-cAMP (100 μM) on ATC-induced cADPR and NAADP formation. **(E)** Effect of Ca^2+^ second messenger inhibitors and cAMP antagonist on ATC-induced GSH formation. **(F-H)** Effect of GSH inhibitor, Diehtyl Maleate (DEM) (50 μM) on ATC-induced formation of cAMP, cADPR and NAADP. **(I)** Inhibitory effect of cAMP antagonists or DEM on ATC-induced insulin secretion. *, P<0.05 versus CON level. #, P<0.05 versus ATC treated level. All data are expressed as the Mean ± SEM.

Treatment with TC analogs increases intracellular GSH, which in turn induces an increase in cAMP levels [35,36]. We examined the effects of ATC treatment on cAMP production and found that ATC induced cAMP production (Fig 3A). The ATC-induced cAMP production was not affected by Ca^2+^ signaling inhibitors, XesC, NED19, or 8-Br-cADPR, suggesting that cAMP generation is located upstream to ATC-induced Ca^2+^ signaling (Fig 3B). Pretreatment with H89 (10 μM), a PKA inhibitor, blocked the ATC-induced Ca^2+^ rise, confirming that cAMP has a role in ATC-induced Ca^2+^ signaling (Fig 3A). To test the possibility that cAMP is a mediator in the ATC-induced production of Ca^2+^ signaling messengers, we measured ATC-induced production of NAADP and cADPR in the absence or presence of a PKA inhibitor or cAMP antagonist. Pretreatment of islets with H89 or Rp-cAMP (100 μM) blocked both the NAADP and cADPR formation that was induced by ATC (Fig 3C and 3D). ATC-induced glutathione synthesis was not affected by cAMP antagonists or Ca^2+^ signaling inhibitors, XesC, NED19, or 8-Br-cADPR (Fig 3E), while cAMP formation was blocked by the glutathione-depleting compound, diethyl maleate (DEM, 50 μM) (Fig 3F). These findings suggest that glutathione induces cAMP formation. Glutathione-depletion by DEM blocked NAADP and cADPR formation (Fig 3G and 3H). Consequently, DEM, as well as H89 and Rp-cAMP, inhibited ATC-induced insulin secretion (Fig 3I).

### ATC induced insulin secretion via the NO-cGMP pathway

**Fig 4:**
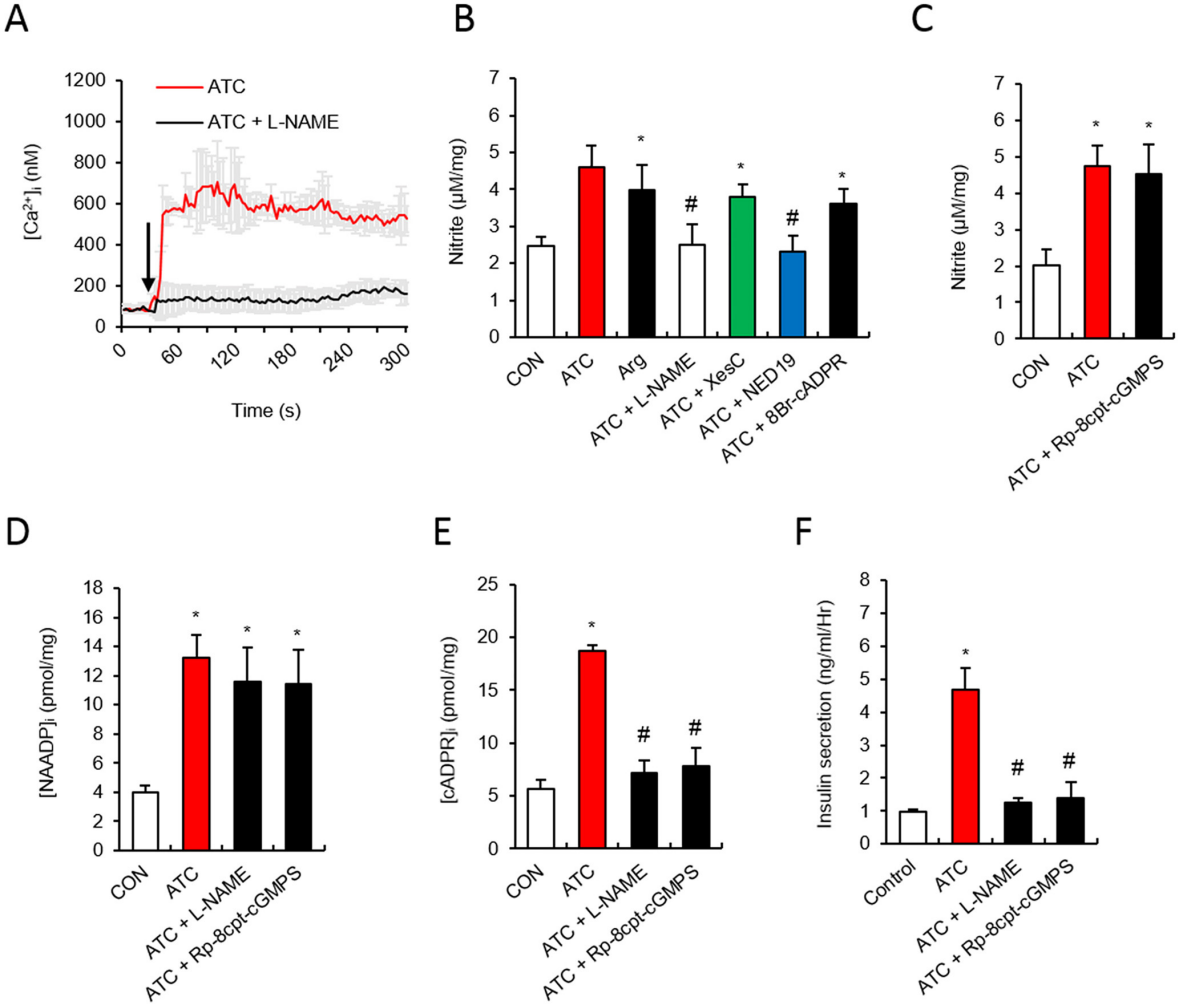
NOS involves in ATC-induced cADPR formation but not in NAADP formation in pancreatic β cell. **(A)** Effect of L-NG-Nitroarginine Methyl Ester (L-NAME) (5 mM) on ATC-induced Ca^2+^ signal. **(B)** Effect of L-NAME and Ca^2+^ second messenger inhibitors on ATC-induced nitrite formation. **(C) E**ffect of cGMP antagonist, (*R*_p_)-8-pCPT-cGMPS (20 μM) on ATC-induced NO formation. **(D and E)** Effect of L-NAME and cGMP antagonist on ATC-induced cADPR and NAADP formation. **(F)** Effect of L-NAME and cGMP antagonists on ATC-induced insulin secretion. *, P<0.05 versus CON level. #, P<0.05 versus ATC treated level. All data are expressed as the Mean ± SEM.

Since arginine stimulates insulin release from pancreatic islets [3], we examined the effects of ATC as an arginine donor in inducing insulin secretion. Pretreatment of cells with L-NAME (5 mM), an inhibitor of NOS, blocked the ATC induced Ca^2+^ rise (Fig 4A). When we measured nitrite as an indicator of NO production, ATC induced a significant rise in nitrite levels when compared to high glucose alone. ATC-induced nitrite levels were comparable to those induced by arginine (Fig 4B). ATC-induced nitrite levels were not affected by pretreatment with XeC or 8-Br-cADPR, while L-NAME and Ned19 blocked nitrite formation, suggesting that NO formation is dependent on NAADP formation (Fig 4B). L-NAME only blocked ATC-induced cADPR formation, leaving the NAADP formation intact (Fig 4D and 4E), indicating that NO production is downstream to NAADP formation, and that NO plays a role in cADPR formation.

NO has been known to activate guanylyl cyclase to produce cGMP [37], which is a second messenger in the production of cADPR [38]. Therefore, it is assumed that the NO-cGMP pathway lies upstream to cADPR formation. To test this assumption, we examined the effects of a cGMP antagonistic analog, Rp-8-pCPT-cGMPS (20 μM), on NAADP and cADPR formation, and found that it blocked only cADPR formation (Fig 4D and 4E). These findings suggest that cGMP plays a role in cADPR formation in ATC-induced Ca^2+^ signaling. L-NAME, as well as Rp-8-pCPT-cGMPS, blocked ATC-induced insulin secretion (Fig 4 F). These data show that the NO-cGMP pathway plays an essential role in the ATC-induced insulin secretion.

### Role of nNOS in ATC-mediated Ca^2+^ signalling

**Fig 5:**
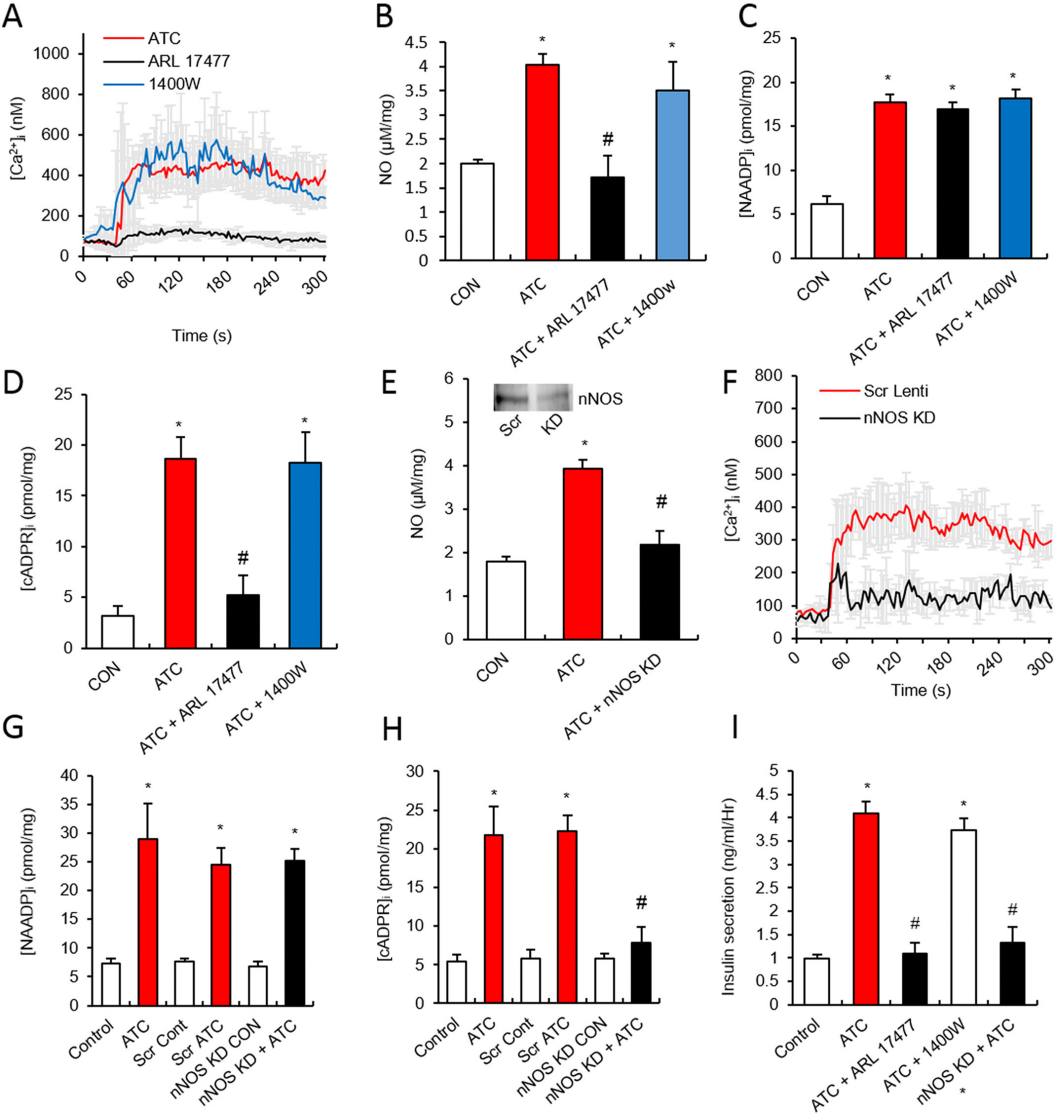
nNOS plays a major role in ATC-induced Ca^2+^ signaling and insulin secretion in pancreatic β cell. **(A)** Effect of nNOS inhibitor, ARL17477 (30 μM) and iNOS inhibitor, 1400W (100 μM) on ATC-induced Ca^2+^ signals. **(B-D)** Effect of nNOS and iNOS inhibitors on ATC-induced NO, cADPR, NAADP formation. **(E)** Effect of nNOS knock down (KD) on ATC-induced NO formation. **(inset)** Representative immunoblots for quantifications of nNOS protein expression in pancreatic β cell after infection with lentiviral particles expressing scrambled or nNOS-specific short hairpin (shRNA). **(F)** Effect of nNOS KD on ATC-induced Ca^2+^ signal. **(G and H)** Effects of nNOS KD on ATC-induced cADPR and NAADP formation. **(I)** Effect of nNOS inhibitors and nNOS KD on ATC-induced insulin secretion. *, P<0.05 versus CON level. #, P<0.05 versus ATC treated level. All data are expressed as the Mean ± SEM.

Since our data showed that L-NAME inhibited ATC-induced Ca^2+^ signalling (Fig 4A), we investigated which isoform(s) of NOS were involved. ARL17477 (30 μM), a nNOS inhibitor, blocked the ATC-induced Ca^2+^ rise, while 1400W (100 μM), an iNOS inhibitor, did not block the ATC-induced Ca^2+^ rise (Fig 5A). The effect of NOS inhibitors on ATC-induced NO formation correlated with our data on Ca^2+^ (Fig 5B). NAADP formation was not affected by NOS inhibitors (Fig 5C), while cADPR formation was completely inhibited by ARL 17477 (Fig 5D). Knockdown of nNOS using lentiviral particles confirmed the pharmacological data, as evidenced by showing that ATC-induced NO formation and Ca^2+^ rise was completely inhibited by nNOS knockdown (Fig 5E and 5F). ATC-induced NAADP formation was not blocked by nNOS knockdown (Fig 5G), whereas cADPR formation was inhibited by knockdown of nNOS (Fig 5H). Finally, the effects of knockdown and pharmacological inhibition of NOS isoforms on ATC-induced insulin release mirrored similar inhibitory patterns seen in Ca^2+^ signalling, cADPR formation, and NO formation (Fig 5I).

### ATC-induced insulin secretion is dependent on Ca^2+^ signalling that is mediated by cADPR, which is produced by CD38

**Fig 6:**
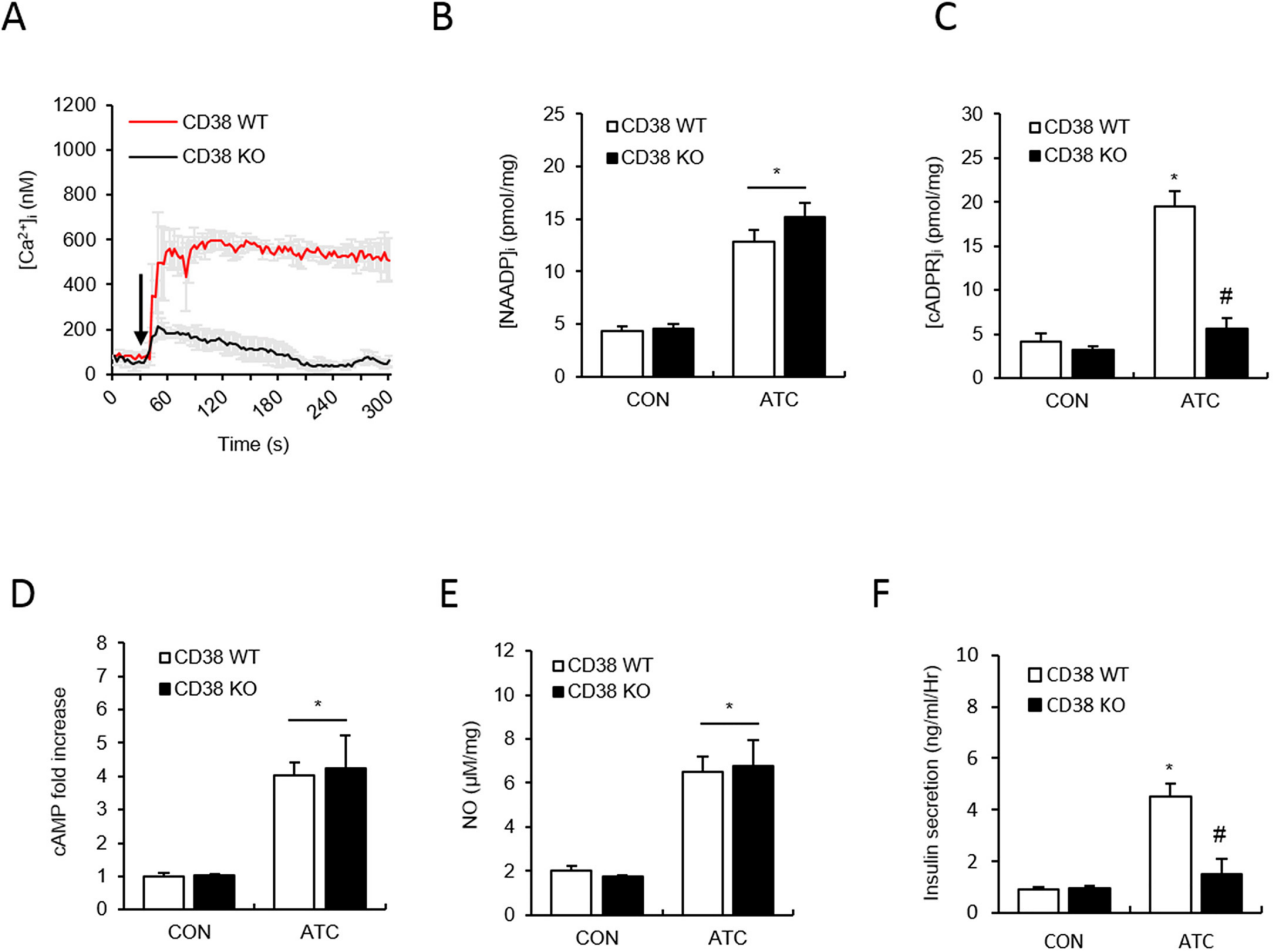
ATC-induced Ca^2+^ signals, cADPR and NAADP production, and insulin secretion in pancreatic β cell from wild-type (WT) and CD38 knock-out (KO) mice. **(A)** Representative tracings of the Ca^2+^ response to ATC in pancreatic β cell prepared from WT and CD38 KO mice. **(B and C)** ATC-stimulated NAADP and cADPR formation in WT and CD38 KO mice. **(D and E)** ATC-stimulated cAMP and NO formation in WT and CD38 KO mice. **(F)** ATC-stimulated insulin secretion in WT and CD38 KO mice. *, P<0.05 versus CON level. #, P<0.05 versus ATC treated level. All data are expressed as the Mean ± SEM.

Since we have shown that OTC enhances CD38 internalization and results in cADPR production [2], we examined whether ATC-mediated Ca^2+^ signals are dependent on CD38 by comparing Ca^2+^ signals in CD38^+/+^ and CD38^-/-^ mice upon treatment with ATC. Islet cells from CD38^-/-^ mice showed markedly reduced levels of sustained Ca^2+^ signals, compared to those from CD38^+/+^ mice (Fig 6A). To further confirm which second messenger was responsible for the difference in ATC-induced increases in Ca^2+^ levels in CD38^+/+^ and CD38^-/-^ mice, we measured NAADP and cADPR levels in both CD38^+/+^ and CD38^-/-^ islets after treatment with ATC. cADPR levels were decreased in CD38^-/-^ islets when compared to wild type islets upon ATC treatment, whereas NAADP levels in CD38^-/-^ islets were the same as those in wild type islets after ATC treatment (Fig 6B and 6C). ATC-induced formation of cAMP and NO, both signalling molecules upstream to CD38, displayed no difference between CD38^+/+^ and CD38^-/-^ islets (Fig 6D and 6E). ATC-induced insulin secretion was decreased in CD38^-/-^ islets (Fig 6F), indicating that CD38-mediated cADPR production plays an important role in ATC-induced insulin secretion.

## Discussion

**Fig 7:**
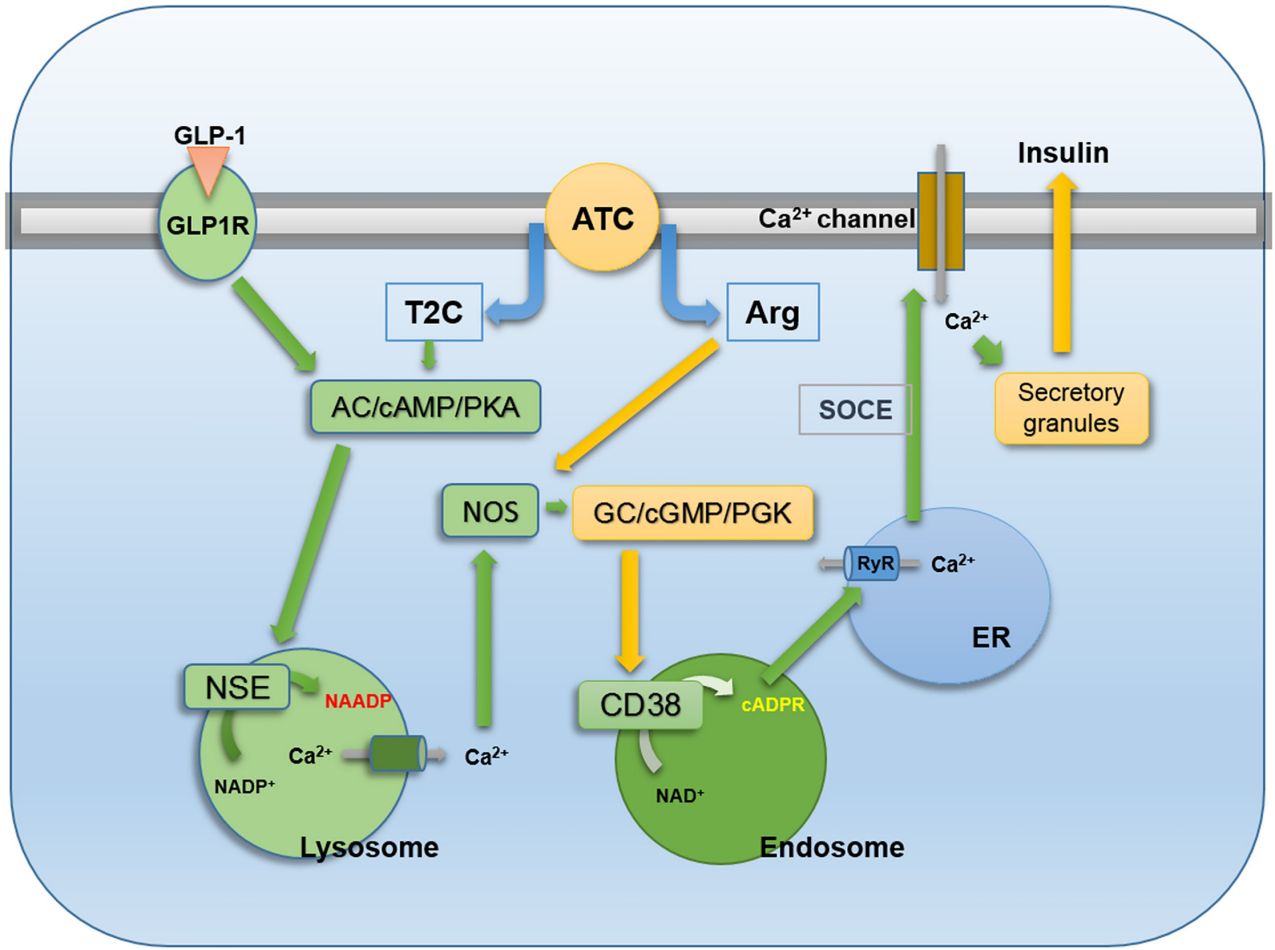
Schematic representation of ATC-induced insulin secretion via cADPR and NAADP production as well as role of NO in pancreatic β cell. Arginine Thiazolidine Carboxylate (ATC) enters and is divided into TC and arginine. TC contributes for Glutathione (GSH) formation, which stimulates adenylyl cyclase, resulting in the production of cAMP. cAMP/PKA activates NSE to produce NAADP, releasing Ca^2+^ from lysosome-related acidic organelles. NAADP-mediated increase of intracellular Ca^2+^ levels results in the activation of NOS. At this moment, arginine is provided as a substrate for Nitric Oxide syntase (NOS). Resulting Nitric Oxide (NO) synthesis activate guanylyl cyclase (GC)/protein kinase G (PKG). PKG activates CD38 to produce cADPR. cADPR-mediated Ca^2+^ release from the ER Ca^2+^ stores. cADPR-mediated Ca^2+^ release regulates the Ca^2+^ influx through store-operated Ca^2+^ entry (SOCE), resulting in insulin secretion in pancreatic β cells. GLP-1, an insulin secretion inducing hormone, also uses similar Ca^2+^ signalling pathway for insulin secretion in pancreatic β cells.

In this study, we showed that a hybrid compound, ATC, induced an increase in Ca^2+^ levels via the sequential production of two Ca^2+^ second messengers: NAADP and cADPR. One component of ATC, TC, increased the production of cAMP, which induced NAADP synthesis. The resulting NAADP-mediated Ca^2+^ rise induced the activation of NOS. NO activated GC to produce cGMP, which led to PKG activation. PKG induced CD38 activation to produce cADPR. The other component of ATC, _L_-arginine, served as a substrate for NOS, augmenting the signals of NAADP-mediated NOS activation (Fig 7). This hybrid compound showed a powerful insulin releasing effect in pancreatic islets when compared to other precursor molecules (Fig 1).

Cysteine is a rate-limiting precursor for the synthesis of GSH and intracellular GSH level is regulated by the availability of cysteine [39]. OTC and TC are the prodrugs of cysteine, and they can raise the plasma concentration of cysteine and the intracellular concentrations of cysteine and GSH [40–42]. We demonstrated OTC is an effective antidiabetic drug by inducing insulin secretion via CD38 internalization, cADPR formation, and subsequent Ca^2+^ rise [2].

This suggested that the cellular reducing system may involve the internalization of CD38 and the formation of cADPR, resulting in insulin secretion. GSH is known to be an activator of adenylyl cyclase [36,43]. As predicted, ATC increased intracellular GSH levels as well as cAMP levels. ATC-induced NAADP and cADPR formation were dependent on cAMP (Fig 3A, 3F and 3G), indicating that cAMP may be upstream to NAADP formation, because cADPR formation is downstream to NAADP-induced Ca^2+^ signaling (Fig 2A and 2F). Therefore, our previous finding that OTC-induced CD38 internalization, resulting in cADPR formation [2], might be attributable to the downstream effect of NAADP formation through GSH-dependent cAMP generation.

Earlier studies showed that OTC is more potent than N-acetylcysteine in increasing reduced glutathione levels [44,45]. It was also found to block airway hyper-responsiveness and inflammation in an asthmatic animal model [46]. Here we found that ATC, a hybrid compound, is more effective in terms of insulin secretion, compared to OTC. ATC was used in the treatment of chronic hepatitis in 1980’s [1]. However, its role in insulin secretion has not been studied. We found that ATC itself induced a rise in Ca^2+^, attributable to the induction of the production of second messengers, NAADP and cADPR, in a sequential manner, which is analogous to that of GLP-1 [21]: When GLP-1 binds to GLP-1R, the stimulatory G protein activates adenylyl cyclase. The resulting production of cAMP induces NAADP synthesis, followed by cADPR formation. The sequential action of these Ca^2+^ second messengers results in Ca^2+^ signals that induce the release of insulin in pancreatic islets. Another analogous feature is the lack of involvement of IP_3_ in both signalling pathways (Fig 2B). Therefore, ATC is likely a physiological insulin secretory hormone mimic. Moreover, ATC supplies _L_-arginine, which is known to have insulin secretory potential [3,4]. This was confirmed by data showing that ATC is more potent in inducing Ca^2+^ signals, the production of Ca^2+^ second messengers, and insulin secretion, when compared to T2C and OTC (Fig 1).

Our data demonstrated that ATC as an arginine provider is a potent NOS activator (Fig 4B). The ATC-mediated NOS activation was found to be NOS isoform-specific in pancreatic islets; ATC activates nNOS preferentially, but not iNOS (Fig 5). Our data showed that NOS is upstream to cADPR formation and downstream to NAADP formation (Fig 5G and 5H), indicating that nNOS plays a role as a linker between the two Ca^2+^ second messengers. Based on our finding that cADPR is produced by CD38 (Fig 6C), NOS is important for the activation of CD38 in cADPR production. The NO-mediated activation of CD38 likely occurs via cGMP/PKG (Fig 4C–4E). Previously, we demonstrated that CD38 is internalized in a PKG-dependent manner through the phosphorylation of myosin heavy chain IIA, resulting in cADPR formation in lymphokine-activated killer (LAK) cells [47]. Since OTC induced CD38 internalization in pancreatic islets [2], ATC might also induce CD38 internalization.

Regarding the question of whether CO, another kind of gaseous transmitter, can induce insulin release, its role as a gaseous transmitter in the insulin secretion pathway has been proved in previous studies as follows: Hemin, the natural substrate for heme oxygenase (HO), increased [Ca^2+^]i transients in β-cells, and Zn-protoporphyrin, a HO inhibitor, inhibited glucose-stimulated insulin release from pancreatic islets [48]. As both CO and NO act on guanylate cyclase to generate cGMP [49], it is interesting to see the causal relationship of the two gaseous transmitters. Crosstalk between the signaling pathways of the two transmitters has been studied: NO and NO-related species induce HO-1 expression [49], suggesting that CO is involved in insulin secretion as an active messenger, and that NO may act via the activation or induction of HO. Regarding the action of H2S, the third gaseous transmitter, on the secretion of insulin from pancreatic β-cells, it has been reported that this gaseous transmitter inhibits insulin release [50,51]. On the other hand, H2S reduces the cellular stress evoked by glucose, possibly through its anti-oxidant properties [52].

Concerning the question of whether ATC additively stimulates insulin release in the presence of simultaneous sulfonylurea (SU) or GLP-1 treatment, our data showed that the combined treatment of ATC with SU or GLP-1 had a higher efficacy than any singular treatment (S5 Fig), suggesting that ATC may use different pathways from those utilized by SU or GLP-1 for insulin secretion. Regarding insulin secretion stimulation potency, ATC and SU were comparable (S5 Fig).

Given that NAADP has demonstrated auto/paracrine functions in vivo and in vitro [53,54], it can be assumed that NAADP released from pancreatic islets upon stimulation with ATC plays a beneficial role by acting on the peripheral tissues, such as adipose tissues. Since NAADP is a Ca^2+^ messenger that is essential for glucose uptake in adipose tissues [55], it is expected that ATC-induced NAADP formation in pancreatic islets results in an ameliorating effect in glucose homeostasis via paracrine functions.

One of the notable findings in this study is the existence of a novel NAADP-synthesizing enzyme other than CD38 as evidenced by data showing that islets from CD38^-/-^ mice showed no impairment in NAADP formation upon ATC treatment, compared to those from wild type mice (Fig 6B). Because CD38 is capable of producing NAADP as well as cADPR [29,56], it is quite interesting to see why two different enzymes participate in ATC-induced insulin secretion in pancreatic islets. β-adrenergic agonist-stimulated cardiomyocytes have also displayed similar NAADP-synthesizing enzymes and CD38 for NAADP and cADPR production, respectively (Gul *et al*. unpublished).

In conclusion, we have demonstrated that ATC, an arginine-containing prodrug of cysteine, induces insulin secretion via Ca^2+^ signalling messenger formation in mice. ATC is a likely physiological agent, because it harnesses the cellular signalling system to increase intracellular Ca^2+^ levels, resulting in insulin secretion. Our results imply that ATC has the potential to be applied as an ideal anti-diabetic drug.

## Supporting Information

S1 Fig The chemical structural formulas of ATC, T2C, Arg and OTC. 1: Oxothiazolidine-4-carboxylicacid (OTC), 2: Thiazolidine-2-carboxylicacid (T2C), 3: Thiazolidine-4-carboxylicacid (T4C), 4: Arginine, 5: Arginine thiazolidine-2-carboxylicacid (ATC), 6: Arginine thiazolidine-4-carboxylic acid (ATC).
(PDF)

S2 Fig Prodrug, Thiazolidine-2-carboxylicacid (T2C) has more effective to produce GSH than Thiazolidine-4-carboxylicacid (T4C) in pancreatic β cell. *, P<0.05 versus CON GSH level. #, P<0.05 versus T2C treated GSH level. All data are expressed as the Mean ± SEM.
(PDF)

S3 Fig ATC-induced cADPR and NAADP production in pancreatic β cell. Islets were treated with/without ATC (100, 400, 700 μM, and 1 μM) and measured cADPR (A) and NAADP (B) levels. *, P<0.05 versus CON cADPR and NAADP level. All data are expressed as the Mean ± SEM.
(PDF)

S4 Fig Effect of RYR inhibitor (20 μM Ryanodine) on ATC-induced Ca^2+^ signals. (PDF)

S5 Fig Effect of ATC, SU, GLP-1 and combined treatment on insulin secretion in pancreatic islets. *, P<0.05 versus CON insulin secretion level. #, P<0.05 versus ATC complex treated level. All data are expressed as the Mean ± SEM.
(PDF)
