# Supplementary material for: Arginine Thiazolidine Carboxylate Stimulates Insulin Secretion through Production of Ca2+-Mobilizing Second Messengers NAADP and cADPR in Pancreatic Islets
Source: PLoS One. 2015 Aug 6;10(8):e0134962. doi: 10.1371/journal.pone.0134962 (PMC4527757; doi:10.1371/journal.pone.0134962)
Supplement: S1 Fig — 1: Oxothiazolidine-4-carboxylicacid (OTC), 2: Thiazolidine-2-carboxylicacid (T2C), 3: Thiazolidine-4-carboxylicacid (T4C), 4: Arginine, 5: Arginine thiazolidine-2-carboxylicacid (ATC), 6: Arginine thiazolidine-4-carboxylic acid (ATC). (PDF) [file pone.0134962.s001.pdf]

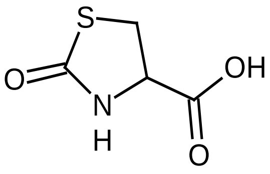

1

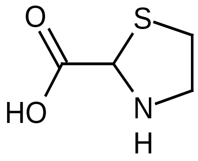

2

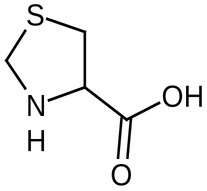

3

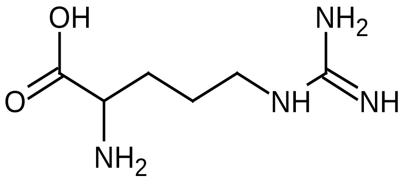

4

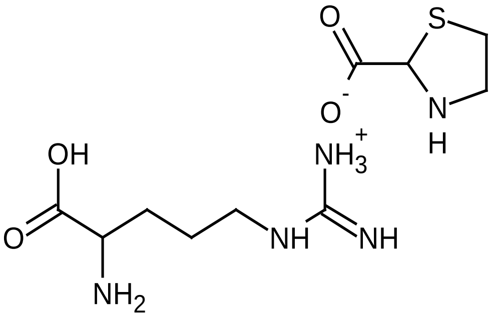

5

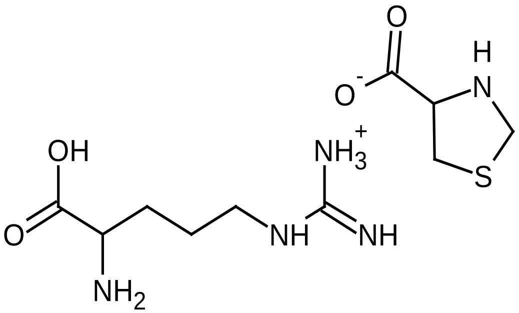

6

S1 Fig. **The chemical structural formulas of ATC, T2C, Arg and OTC.** 1: Oxothiazolidine-4-carboxylic acid (OTC), 2: Thiazolidine-2-carboxylic acid (T2C), 3: Thiazolidine-4-carboxylic acid (T4C), 4: Arginine, 5: Arginine thiazolidine-2-carboxylic acid (ATC), 6: Arginine thiazolidine-4-carboxylic acid (ATC)
