# Supplementary material for: Arginine Thiazolidine Carboxylate Stimulates Insulin Secretion through Production of Ca2+-Mobilizing Second Messengers NAADP and cADPR in Pancreatic Islets
Source: PLoS One. 2015 Aug 6;10(8):e0134962. doi: 10.1371/journal.pone.0134962 (PMC4527757; doi:10.1371/journal.pone.0134962)
Supplement: S2 Fig — *, P<0.05 versus CON GSH level. #, P<0.05 versus T2C treated GSH level. All data are expressed as the Mean ± SEM. (PDF) [file pone.0134962.s002.pdf]

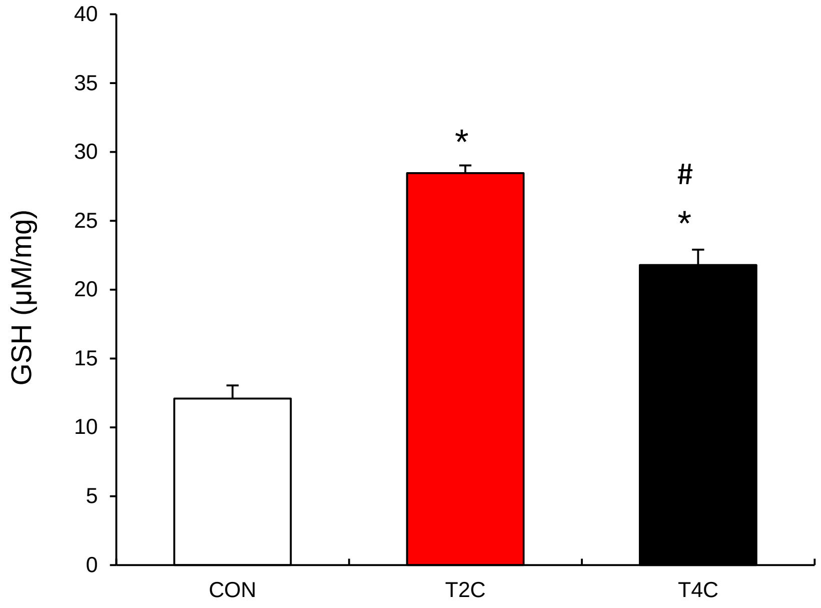

S2 Fig. Prodrug, Thiazolidine-2-carboxylic acid (T2C) has more effective to produce GSH than Thiazolidine-4-carboxylic acid (T4C) in pancreatic β cell. *, P<0.05 versus CON GSH level. #, P<0.05 versus T2C treated GSH level. All data are expressed as the Mean ± SEM.
