# Supplementary material for: Arginine Thiazolidine Carboxylate Stimulates Insulin Secretion through Production of Ca2+-Mobilizing Second Messengers NAADP and cADPR in Pancreatic Islets
Source: PLoS One. 2015 Aug 6;10(8):e0134962. doi: 10.1371/journal.pone.0134962 (PMC4527757; doi:10.1371/journal.pone.0134962)
Supplement: S3 Fig — Islets were treated with/without ATC (100, 400, 700 μM, and 1 μM) and measured cADPR (A) and NAADP (B) levels. *, P<0.05 versus CON cADPR and NAADP level. All data are expressed as the Mean ± SEM. (PDF) [file pone.0134962.s003.pdf]

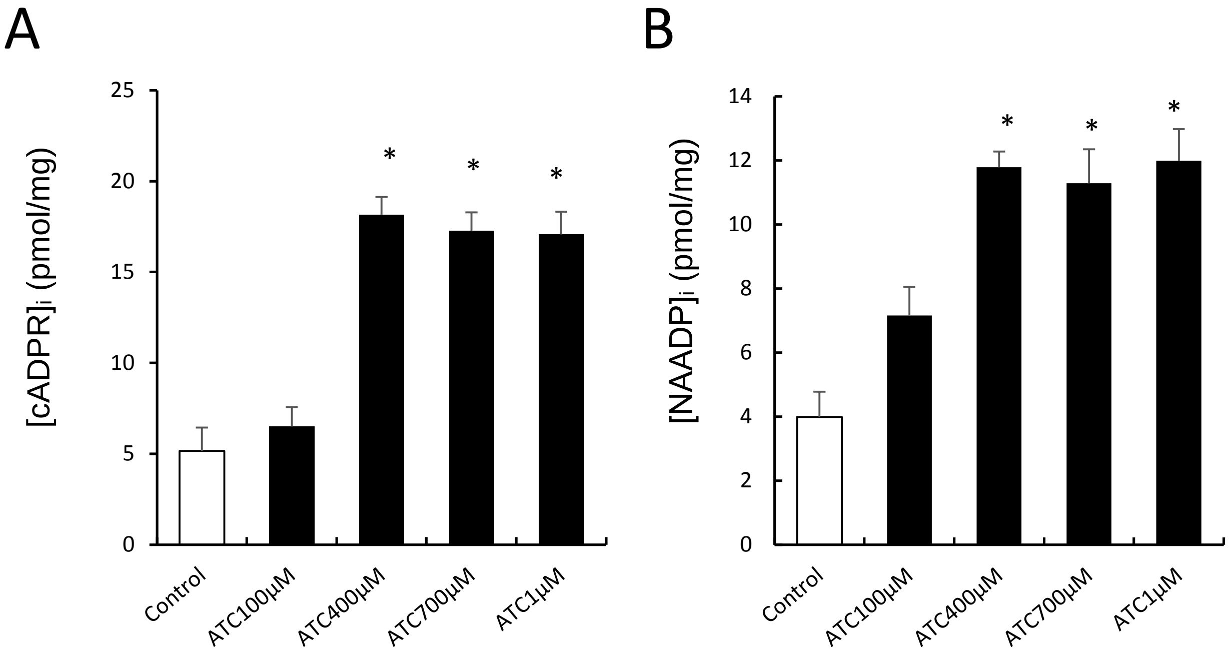

S3 Fig. ATC-induced cADPR and NAADP production in pancreatic β cell. Islets were treated with/without ATC(100, 400, 700 μM, and 1 μM) and measured cADPR (A) and NAADP (B) levels.*, P<0.05 versus CON cADPR and NAADP level. All data are expressed as the Mean ± SEM.
