# Supplementary figures and images for: Arginine Thiazolidine Carboxylate Stimulates Insulin Secretion through Production of Ca2+-Mobilizing Second Messengers NAADP and cADPR in Pancreatic Islets
Source: PLoS One. 2015 Aug 6;10(8):e0134962. doi: 10.1371/journal.pone.0134962 (PMC4527757; doi:10.1371/journal.pone.0134962)

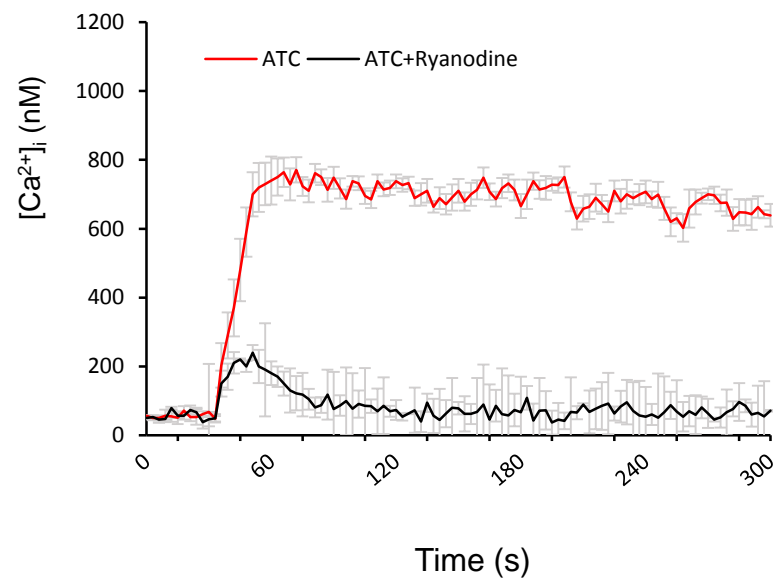

**S4 Fig. Effect of RYR inhibitor (20  $\mu$ M Ryanodine) on ATC-induced  $Ca^{2+}$  signals.**

Supplement: S4 Fig — (PDF) [file pone.0134962.s004.pdf]
