# Supplementary material for: Arginine Thiazolidine Carboxylate Stimulates Insulin Secretion through Production of Ca2+-Mobilizing Second Messengers NAADP and cADPR in Pancreatic Islets
Source: PLoS One. 2015 Aug 6;10(8):e0134962. doi: 10.1371/journal.pone.0134962 (PMC4527757; doi:10.1371/journal.pone.0134962)
Supplement: S5 Fig — *, P<0.05 versus CON insulin secretion level. #, P<0.05 versus ATC complex treated level. All data are expressed as the Mean ± SEM. (PDF) [file pone.0134962.s005.pdf]

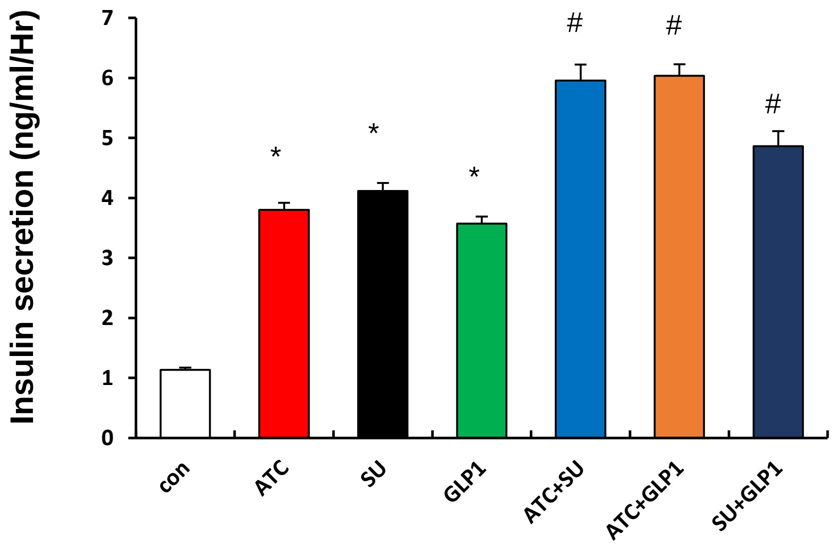

S5 Fig. Effect of ATC, SU, GLP-1 and combined treatment on insulin secretion in pancreatic islets. *, P<0.05 versus CON insulin secretion level. #, P<0.05 versus ATC complex treated level. All data are expressed as the Mean ± SEM.
